# Early Postintubation Hemodynamic Changes Following Remimazolam or Propofol Induction With Simultaneous Bolus Remifentanil in Total Hip Arthroplasty: A Retrospective Observational Study

**DOI:** 10.7759/cureus.111755

**Published:** 2026-06-29

**Authors:** Nobuhide Sasaki, Tomoharu Shakuo, Shinichiro Morotomi, Shota Tanimoto, Ken Hirai, Sae Ono, Kenichi Takahashi, Takuya Dosei, Yusuke Ishida, Atsunori Sakamoto, Megumi Hashimoto, Kenji Shida

**Affiliations:** 1 Department of Anesthesiology, Showa Medical University Northern Yokohama Hospital, Yokohama, JPN; 2 Department of Anesthesiology, Showa Medical University School of Medicine, Tokyo, JPN

**Keywords:** anesthesia induction, hemodynamics, mean arterial pressure, postintubation hypotension, propofol, remifentanil, remimazolam, total hip arthroplasty

## Abstract

Background

Early postintubation hypotension is a clinically relevant concern during anesthesia induction. During induction, whether remimazolam provides a hemodynamic advantage over propofol remains unclear. Here, we aimed to compare postintubation hypotension between remimazolam-based and propofol-based anesthesia induction.

Methods

In this single-center retrospective study, we included patients undergoing elective total hip arthroplasty. Anesthesia was induced using a standardized simultaneous-bolus protocol with remifentanil 3 μg/kg and either remimazolam 0.2 mg/kg or propofol 1.0 mg/kg. The primary outcome was the occurrence of at least one one-minute epoch with a mean arterial pressure (MAP) <65 mmHg within five minutes of postintubation. Sensitivity outcomes were defined using a MAP threshold of <60 mmHg, a decrease in MAP of ≥30% from preinduction MAP, and a 0-3 min postintubation window. Secondary outcomes included percentage change in MAP from hypnotic administration to five minutes postintubation; MAP trajectories, systolic blood pressure, diastolic blood pressure, heart rate, and bispectral index; minimum MAP during induction; time-weighted average MAP below 65 mmHg; maximum increase in systolic blood pressure; and vasopressor use. Analyses were performed using cases with sufficient induction time-series data to reconstruct the predefined time points.

Results

Of the 190 screened cases, 59 were included in the analysis: 33 in the remimazolam group and 26 in the propofol group. Early postintubation hypotension occurred in 19 patients (57.6%) in the remimazolam group and 14 patients (53.8%) in the propofol group. No significant between-group difference was observed in the primary outcome, and sensitivity analyses yielded concordant findings. In the exploratory multivariable logistic regression analysis, hypnotic agent selection was not significantly associated with hypotension. Older age and lower preinduction MAP were associated with a higher risk of hypotension. No significant between-group differences in secondary outcomes were noted. No refractory or life-threatening hypotension was observed.

Conclusions

Under a standardized simultaneous-bolus induction protocol with remifentanil, no significant difference in early postintubation hypotension was observed between patients receiving remimazolam and those receiving propofol. Older age and lower preinduction MAP were associated with hypotension, whereas hypnotic agent selection was not. Clinically relevant differences and selection bias cannot be excluded owing to the small analyzable cohort, wide confidence intervals, nonrandom hypnotic selection, and the exclusion of many screened cases because of insufficient induction records for analysis.

## Introduction

Perioperative hypotension has been associated with increased risks of acute kidney injury, myocardial injury, cardiovascular and cerebrovascular events, and mortality. The recent PeriOperative Quality Initiative (POQI) consensus statement recommends maintaining an intraoperative mean arterial pressure (MAP) of ≥60 mmHg in at-risk patients [1]. In addition, observational studies have reported associations between MAP values below 65 mmHg and adverse postoperative outcomes, particularly when hypotension is more severe or more prolonged [2-5]. Notably, the extent of blood pressure (BP) decline during anesthesia induction may depend on the anesthetic agents used and the selected dosing strategy. Propofol can promote hypotension through vasodilation and myocardial depression, whereas remimazolam, an intravenous benzodiazepine anesthetic, is generally considered to result in a more stable hemodynamic profile. However, whether remimazolam reduces induction-related hypotension compared with propofol has not been consistently demonstrated across patient populations or dosing approaches [6-10]. For example, a randomized trial reported no reduction in hypotension with remimazolam compared with propofol in patients aged ≥80 years [7]. Another study using target-controlled propofol administration also reported no significant difference in postinduction BP decline [8].

Remifentanil is commonly coadministered to attenuate the sympathetic response to laryngoscopy. While the pharmacodynamics of remimazolam and the hemodynamic effects of remifentanil bolus administration have been described previously [11-13], evidence directly comparing remimazolam and propofol administered as simultaneous single boluses with remifentanil remains limited.

Other reported risk factors for induction-related hypotension include older age, lower preinduction BP, angiotensin-converting enzyme inhibitor or angiotensin receptor blocker (ACEI/ARB) therapy, and β-blocker therapy [14]. These factors are clinically relevant in patients undergoing total hip arthroplasty (THA), in whom peri-induction hemodynamic stability is particularly important. This study aimed to compare early hemodynamic responses during anesthesia induction between remimazolam- and propofol-based induction in patients undergoing THA, with each hypnotic administered as a simultaneous single bolus with remifentanil.

## Materials and methods

Study design and participants

This single-center retrospective observational study was approved by the Institutional Review Board of Showa Medical University (No. 2024-251-B). The study was conducted at Showa Medical University Northern Yokohama Hospital from January 2024 to August 2025 and in accordance with the Declaration of Helsinki. The requirement for written informed consent was waived by the institutional review board because of the retrospective design and the use of anonymized data, with an opt-out option provided on the institutional website. The reporting follows the Strengthening the Reporting of Observational Studies in Epidemiology (STROBE) guidelines.

No a-priori sample size calculation was performed because this was an exploratory retrospective observational study that included all eligible cases with analyzable induction time-series records during the predefined study period. The inclusion criteria were age 20-90 years, American Society of Anesthesiologists Physical Status (ASA-PS) classification I or II, and elective THA. The exclusion criteria were emergency surgery, cardiovascular disease other than hypertension, pregnancy, severe hepatic dysfunction, dialysis, prior central nervous system disease, relevant drug allergy, severe dyslipidemia, obesity defined as body mass index (BMI) ≥30 kg/m², and opting out of the study. For patients who underwent repeat procedures, only the first procedure was analyzed. During the study period, elective THA at our institution was routinely performed under general anesthesia according to the institutional perioperative pathway. Neuraxial anesthesia or peripheral nerve blocks, when used, were primarily administered as adjuncts for perioperative analgesia rather than as sole anesthetic techniques. Therefore, this retrospective study included patients undergoing THA under general anesthesia and focused on early hemodynamic changes during standardized anesthesia induction.

Standardized induction protocol

The induction protocol was developed at our institution; a propofol-based method was iteratively optimized before remimazolam became available in 2020 and subsequently revised to incorporate considerations related to remimazolam pharmacology. The protocol is applied to many elective procedures, including THA, but not to high-risk cases such as cardiac surgery.

In this protocol, anesthesia induction was performed using a standardized simultaneous-bolus technique. Either remimazolam 0.2 mg/kg or propofol 1.0 mg/kg was administered intravenously as the hypnotic agent, followed immediately by rocuronium 1.0 mg/kg and then remifentanil 3 μg/kg. The hypnotic dose was calculated according to body weight and was not titrated based on bispectral index (BIS) values. BIS was used to monitor anesthetic depth and guide subsequent anesthetic management, but not for determining the initial hypnotic dose. Thus, the hypnotic agent, rocuronium, and remifentanil were administered as rapid consecutive boluses rather than as separate titrated infusions. The time of hypnotic administration was defined as Ts. After an interval intended to allow sufficient neuromuscular relaxation, usually within one minute, tracheal intubation was performed following several manual mask ventilations in most cases. The completion of endotracheal tube insertion and cuff inflation was defined as T0, and T1-T5 were defined as one to five minutes after T0, respectively.

All induction drugs were administered manually as intravenous bolus injections according to routine clinical practice. The exact injection speeds and time intervals between hypnotic administration, rocuronium administration, remifentanil administration, and tracheal intubation were not systematically recorded in the source records. During induction, acetated Ringer's solution was generally administered rapidly by gravity with the infusion clamp fully open, unless clinically contraindicated.

MAP, systolic BP (SBP), diastolic BP (DBP), heart rate (HR), and BIS were recorded at each time point and are denoted as MAP_Tk, SBP_Tk, DBP_Tk, HR_Tk, and BIS_Tk, respectively (where Tk represents Ts, T0, T1, T2, T3, T4, or T5). BP was monitored using automated noninvasive oscillometric measurements during induction. Measurements were obtained according to routine clinical practice and extracted at the predefined time points.

Automated noninvasive BP measurements were obtained at approximately one-minute intervals during the induction period. Desflurane administration at 6% was initiated once BIS had recovered to ≥61 after hypnotic administration and was adjusted to maintain BIS at 40-60. During induction, ephedrine 4 mg or phenylephrine 0.05 mg was administered if the MAP was ≤60 mmHg. The choice of hypnotic agent was not randomized. During the study period, both propofol and remimazolam were available as intravenous hypnotic agents for general anesthesia induction at our institution. No formal institutional criteria or predefined algorithm mandated the use of either agent. The final choice of remimazolam or propofol was made by the attending anesthesiologist according to routine clinical practice. Once the hypnotic agent was selected, the induction dose was administered according to the standardized protocol described above.

Outcomes

The primary outcome was any one-minute epoch with MAP <65 mmHg (Hypo65) during T0-T5. Sensitivity analyses were performed using the following alternative definitions: (i) MAP <60 mmHg, (ii) a ≥30% decrease in MAP from Ts, and (iii) the T0-T3 postintubation window. The T0-T5 window was selected to capture the early postintubation period, during which marked hemodynamic changes were expected following the simultaneous-bolus induction protocol, while minimizing the influence of subsequent intraoperative factors.

The secondary outcomes were as follows: (a) percentage change in MAP from Ts to T5, calculated as (MAP_T5 − MAP_Ts) / MAP_Ts × 100; (b) trajectories of MAP, SBP, DBP, HR, and BIS from T0 to T5; (c) minimum MAP during induction, defined as the absolute minimum MAP from T0 to T5, and maximal decrease in MAP from Ts; (d) time-weighted average (TWA) MAP <65 mmHg, defined as the mean per minute shortfall <65 mmHg over T0-T5; (e) sympathetic response, defined as maximum increase in SBP during T0-T5 relative to SBP_Ts; and (f) vasopressor use, defined as ephedrine or phenylephrine administration during T0-T5. Baseline variables included age, sex, BMI, ASA-PS, antihypertensive therapy, ACEI/ARB therapy, and MAP_Ts.

Statistical analysis

Continuous variables are reported as mean ± standard deviation or median with interquartile range, as appropriate. Categorical variables are reported as n (%). Welch's t-test or the Wilcoxon rank-sum test was used for between-group comparisons of continuous variables, as appropriate. Fisher's exact test was used for between-group comparisons of categorical variables. The primary outcome was compared using Fisher's exact test, and the risk ratio (RR), risk difference (RD), and odds ratio (OR) with 95% confidence intervals (CIs) were reported. Statistical significance was set at a two-sided α level of 0.05.

The multivariable logistic regression model for Hypo65 included hypnotic agent (reference: propofol), age, MAP_Ts, and use of any antihypertensive therapy. A sensitivity model replacing any antihypertensive therapy with ACEI/ARB therapy was also performed. For logistic regression analyses, adjusted ORs with 95% CIs were reported. The multivariable logistic regression analysis was performed as an exploratory adjusted analysis.

For exploratory time-point comparisons of MAP, SBP, DBP, HR, and BIS, Welch's t tests were performed at each postintubation time point from T0 to T5. Because these analyses were exploratory, unadjusted test statistics and p-values were reported descriptively. Holm adjustment was applied within each variable across the six postintubation time points to assess whether any time-point differences remained statistically significant after correction for multiple comparisons. No adjustment was applied across different variables. No longitudinal or mixed-effects model was fitted, and vasopressor administration during the T0-T5 was not modeled as a time-varying factor. Therefore, these time-point comparisons were considered descriptive analyses. All statistical analyses were performed using JMP® Student Edition version 18.2.2 (SAS Institute Inc., Cary, NC, USA).

Missing data

Analyses were performed using complete cases for the variables required for each analysis. For the primary outcome and predefined time-series outcomes, cases were required to have analyzable induction time-series records sufficient to reconstruct the relevant predefined time points from T0 to T5. Cases in which these time points could not be reconstructed from the source records were excluded from the corresponding analyses.

Missing or non-analyzable induction records resulted from incomplete electronic records, documentation omissions, automated noninvasive BP measurement failures, or extended measurement intervals, and BIS sensor drop-off or artifacts. The largest exclusion category comprised cases for which no analyzable induction time-series records were available. These cases reflected the absence of source data required to reconstruct the predefined induction time points rather than isolated missing values within otherwise complete records.

Because the primary outcome and time-series variables could not be determined for these cases, imputation was not performed. Missingness may have been related to routine documentation practices, workflow, device or sensor performance, or time-period effects. Therefore, the potential for selection bias and reduced generalizability due to missing data is addressed in the limitations section.

## Results

Study cohort and baseline characteristics

**Figure 1:**
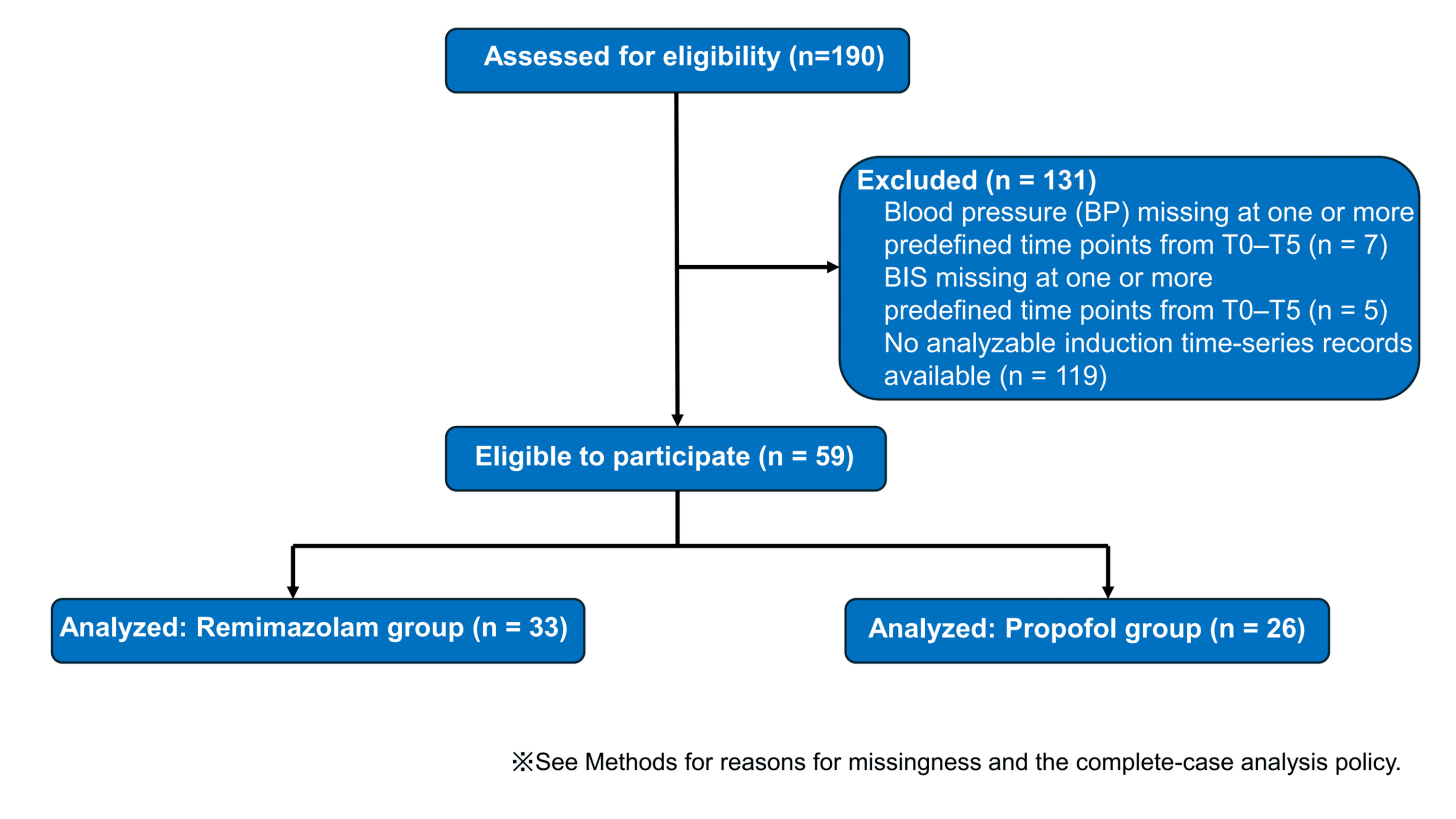
Flow diagram of the study population Flow diagram depicting the inclusion of patients undergoing total hip arthroplasty. Boxes indicate the numbers of records screened, excluded (along with the reasons), and included in the remimazolam and propofol groups. The final study population for the primary endpoint comprised 33 patients in the remimazolam group and 26 in the propofol group (n = 59). The numbers at each node represent the numbers of cases after applying the inclusion and exclusion criteria.

Of the 190 screened cases, 131 were excluded because the predefined induction time-series data required for analysis could not be reconstructed from the source records. The reasons for exclusion were missing BP data at one or more time points from T0 to T5 (n = 7), missing BIS data at one or more time points from T0 to T5 (n = 5), and no analyzable induction time-series records available (n = 119). The last category comprised cases in which time-stamped induction records were not available in a format that permitted reconstruction of the predefined T0-T5 hemodynamic and BIS values. Because the required time-point and outcome data were unavailable in most excluded cases, formal comparison of induction hemodynamics and hypotension outcomes between included and excluded patients was not feasible. Ultimately, 59 cases were included in the analysis: 33 in the remimazolam group and 26 in the propofol group (Figure 1).

Baseline characteristics, including age, BMI, sex, the proportion of patients with ASA-PS II, and the use of ACEI/ARB therapy or other antihypertensive agents, were generally comparable between the groups (Table 1). MAP_Ts was numerically lower in the remimazolam group than in the propofol group (101.45 ± 12.82 mmHg vs. 108.81 ± 17.36 mmHg; mean difference, -7.35 mmHg; 95% CI, -15.55 to 0.85; Cohen's d, -0.49), although the difference was not statistically significant (Welch's t test, t = -1.81, df = 44.67, p = 0.078). None of the patients in either group used β-blockers.

**Table 1:** Baseline characteristics Baseline demographic and clinical characteristics at preinduction (Ts). Continuous variables are summarized as mean ± SD, and categorical variables are summarized as n (%). The n values in the column headings indicate the total number of patients in each group. For categorical variables, n (%) indicates the number and percentage of patients with the corresponding characteristic; percentages were calculated using the total number of patients in each group as the denominator. SMDs were calculated based on the group means and SDs (Remimazolam − Propofol). Continuous and categorical variables were compared using Welch's t-test and Fisher's exact test (two-sided), respectively. The baseline MAP at Ts is the preinduction value. ACEI - angiotensin-converting enzyme inhibitor; ARB - angiotensin II receptor blocker; ASA - American Society of Anesthesiologists physical status; BMI - body mass index; MAP - mean arterial pressure; SMD - standardized mean difference; SD - standard deviation.

Characteristic	Remimazolam (n=33)	Propofol (n=26)	SMD	p-value
Age (yr), mean ± SD	69.21 ± 11.09	66.46 ± 9.50	0.27	—
BMI (kg/m²), mean ± SD	23.01 ± 2.89	23.92 ± 3.32	-0.29	—
MAP_Ts (mmHg), mean ± SD	101.45 ± 12.82	108.81 ± 17.36	-0.48	0.078
ASA II, No. (%)	23 (69.7%)	18 (69.2%)	—	1
ACEI/ARB use, No. (%)	7 (21.2%)	6 (23.1%)	—	1
β-blocker use, No. (%)	0 (0%)	0 (0%)	—	NA
Other antihypertensives, No. (%)	4 (12.1%)	8 (30.8%)	—	0.11
Male, No. (%)	2 (6.1%)	6 (23.1%)	—	0.12

Primary outcome

The incidence of Hypo65 was 57.6% (19/33) in the remimazolam group and 53.8% (14/26) in the propofol group; no significant difference was observed between the groups (Fisher's exact test, two-sided p = 0.798; risk ratio, 1.07; 95% CI, 0.67-1.70; risk difference, 3.73%; 95% CI, -21.80% to 29.25%; odds ratio, 1.16; 95% CI, 0.41-3.27; Table 2). Sensitivity analyses using a 60 mmHg threshold, a ≥30% decrease from Ts, and the T0-T3 window also showed no significant between-group differences (Table 3).

**Table 2:** Primary outcome The primary endpoint was defined as an MAP of <65 mmHg at any time point from T0 to T5 (intubation to five minutes post-intubation; Hypo65). P-values were derived using Fisher's exact test (two-sided). Risk metrics are presented with 95% CIs. CI - confidence interval; MAP - mean arterial pressure; OR - odds ratio; RD - risk difference (Remimazolam - Propofol); RR - risk ratio

Outcome	Remimazolam	Propofol	p (Fisher)	RR (95% CI)	RD (95% CI)	OR (95% CI)
Hypotension (MAP<65 mmHg, any T0-T5)	19/33 (57.6%)	14/26 (53.8%)	0.8	1.07 (0.67-1.70)	+3.73% (-21.80% to +29.25%)	1.16 (0.41-3.27)

**Table 3:** Sensitivity analyses for binary hypotension endpoints Alternative definitions of intrainduction hypotension are as follows: Hypo60 (MAP < 60 mmHg at any time from T0 to T5), HypoRel30 (≥30% reduction from Ts at any time from T0 to T5), and Hypo65_T3 (MAP <65 mmHg within T0-T3). P-values were determined using Fisher's exact test (two-sided). The risk metrics are presented with 95% CIs. These were prespecified sensitivity analyses, and no multiplicity adjustment was applied. T0-T5, intubation to five minutes thereafter; Ts, baseline immediately before induction; CI - confidence interval; MAP - mean arterial pressure; OR - odds ratio; RD - risk difference (Remimazolam − Propofol); RR - risk ratio

Outcome (definition)	Remimazolam	Propofol	p (Fisher)	RR (95% CI)	RD (95% CI)	OR (95% CI)
Hypo60 (MAP<60 mmHg, any T0-T5)	13/33 (39.4%)	10/26 (38.5%)	1	1.02 (0.54-1.95)	+0.93% (-24.12% to +25.98%)	1.04 (0.36-2.99)
HypoRel30 (≥30% drop from Ts)	26/33 (78.8%)	21/26 (80.8%)	1	0.98 (0.75-1.26)	-1.98% (-22.57% to +18.61%)	0.88 (0.24-3.19)
Hypo65_T3 (T0-T3 window)	16/33 (48.5%)	12/26 (46.2%)	1	1.05 (0.61-1.81)	+2.33% (-23.32% to +27.98%)	1.10 (0.39-3.08)

Multivariable analysis

The multivariable logistic regression model was statistically significant overall (likelihood-ratio χ² = 25.22, df = 4, p < 0.0001). Hypnotic agent was not significantly associated with Hypo65 after adjustment (remimazolam vs. propofol (reference): adjusted odds ratio, 0.53; 95% CI, 0.12-2.08; p = 0.37). Older age was associated with higher odds of Hypo65 (adjusted odds ratio per one year increase, 1.16; 95% CI, 1.07-1.28; p < 0.001), whereas higher MAP_Ts was associated with lower odds of Hypo65 (adjusted odds ratio per 1 mmHg increase, 0.93; 95% CI, 0.88-0.98; p = 0.002). Any antihypertensive use was not significantly associated with Hypo65 (adjusted odds ratio, 1.06; 95% CI, 0.22-5.13; p = 0.94).

The lack-of-fit test showed no evidence of poor model fit (χ² = 52.96, df = 53, p = 0.476). The sensitivity model replacing any antihypertensive use with ACEI/ARB therapy yielded similar results (Table 4).

**Table 4:** Multivariable logistic regression analysis (primary endpoint: Hypo65) aORs for Hypo65 were determined using a logistic regression model that included group (Remimazolam vs Propofol (reference)), age (per one year), MAP_Ts (per 1 mmHg), and use of any antihypertensive medication (Yes vs No (reference)). β-blocker use was 0 in both groups and was excluded a priori. The model fit was evaluated using the LOF test (p = 0.48), which indicated no evidence of lack of fit. Two-sided p-values are reported. Abbreviations. aOR, adjusted odds ratio; CI, confidence interval; MAP_Ts, baseline MAP at Ts; LOF, lack of fit.

Predictor (Remi vs Prop)	Adjusted OR	95% CI	p-value
Group	0.53	0.12-2.08	0.37
Age (per 1 yr)	1.16	1.07-1.28	<0.001
MAP_Ts (per 1 mmHg)	0.93	0.88-0.98	0.002
Any antihypertensive use (Yes vs No (reference))	1.06	0.22-5.13	0.94

Secondary outcomes

Vasopressor use and summary hemodynamic outcomes

Vasopressor use during T0-T5 was more frequent in the remimazolam group than in the propofol group, but the difference was not statistically significant (27.3% (9/33) vs. 11.5% (3/26); Fisher's exact test, p = 0.196; risk ratio, 2.36; 95% CI, 0.71-7.86; risk difference, 15.7%; 95% CI, -3.8% to 35.3%; odds ratio, 2.88; 95% CI, 0.69-11.97).

The percentage change in MAP from Ts to T5 did not differ significantly between the groups; the between-group difference was -2.50 percentage points for remimazolam versus propofol (95% CI, -8.66 to 3.66; Welch's t test, t = -0.82, df = 43.67, p = 0.417). TWA MAP below 65 mmHg during T0-T5 was low in the remimazolam and propofol groups and did not differ significantly between the groups (0.60 (0.00-5.30) mmHg vs. 0.20 (0.00-3.50) mmHg; Wilcoxon rank-sum test, S = 751.5, Z = -0.45, p = 0.655).

The maximum MAP decrease from Ts did not differ significantly between the groups (mean difference, -1.61 mmHg; 95% CI, -9.36 to 6.14; Welch's t test, t = -0.42, df = 45.87, p = 0.678). The maximum increase in SBP during T0-T5 was also similar between the groups (mean difference, -2.97 mmHg; 95% CI, -18.32 to 12.39; Welch's t test, t = -0.39, df = 53.40, p = 0.700). Additionally, minimum MAP during induction did not differ significantly between the remimazolam and propofol groups (62 (52-68) mmHg vs. 63.5 (56-79) mmHg; Z = -1.34; p = 0.18; Table 5).

**Table 5:** Secondary outcomes during induction Summary of secondary outcomes during induction (T0-T5). P-value for vasopressor use was determined using Fisher's exact test. Continuous endpoints were compared using Welch’s t test or the Wilcoxon rank-sum test, as appropriate. For Welch-tested continuous outcomes, effect estimates are reported as mean differences (Remimazolam − Propofol) with 95% CIs. TWA MAP below 65 mmHg and minimum MAP during induction are presented as median (IQR) and were compared using the Wilcoxon rank-sum test. Risk metrics (RR, RD, and OR) are shown only for the binary endpoint of vasopressor use. CI - confidence interval; IQR - interquartile range; MAP - mean arterial pressure; OR - odds ratio; RD - risk difference; RR - risk ratio; SBP - systolic blood pressure; TWA - time-weighted average

Outcome	Group summaries	p-value	Statistical test	RR (95% CI)	RD (95% CI)	OR (95% CI)
Vasopressor use during T0-T5	9/33 (27.3%) vs 3/26 (11.5%)	0.196	Fisher's exact test	2.36 (0.71-7.86)	+15.7% (-3.8% to +35.3%)	2.88 (0.69-11.97)
MAP percentage change from Ts to T5	-2.50% (-8.66 to +3.66)	0.42	Welch's t-test	—	—	—
TWA MAP below 65 mmHg during T0-T5, mmHg, median (IQR)	0.60 (0.00-5.30) vs 0.20 (0.00-3.50)	0.65	Wilcoxon rank-sum test	—	—	—
Maximum MAP decrease from Ts, mmHg	-1.61 (-9.36 to +6.14)	0.68	Welch's t-test	—	—	—
Maximum SBP increase during T0-T5, mmHg	-2.97 (-18.32 to +12.39)	0.7	Welch's t-test	—	—	—
Minimum MAP during induction, mmHg, median (IQR)	62 (52-68) vs 63.5 (56-79)	0.18	Wilcoxon rank-sum test	—	—	—

Exploratory time-point comparisons

In exploratory unadjusted time-point comparisons, MAP was numerically lower in the remimazolam group at several postintubation time points. The differences were not statistically significant at T0 (mean difference, -5.17 mmHg; t = -0.98, df = 43.48, p = 0.331) and T1 (mean difference, -6.04 mmHg; t = -1.24, df = 46.70, p = 0.222). Unadjusted differences were observed at T2 (mean difference, -9.01 mmHg; t = -2.04, df = 40.35, p = 0.048), T3 (mean difference, -11.89 mmHg; t = -2.53, df = 33.86, p = 0.016), T4 (mean difference, -9.69 mmHg; t = -2.34, df = 33.48, p = 0.025), and T5 (mean difference, -7.22 mmHg; t = -2.25, df = 40.67, p = 0.030). However, these differences were not statistically significant after Holm adjustment.

Similarly, unadjusted SBP comparisons showed no statistically significant differences at T0 (mean difference, -4.47 mmHg; t = -0.56, df = 45.22, p = 0.579) and T1 (mean difference, -5.46 mmHg; t = -0.83, df = 47.48, p = 0.409). Unadjusted differences were observed at T2 (mean difference, -12.42 mmHg; t = -2.11, df = 42.06, p = 0.041) and T3 (mean difference, -14.58 mmHg; t = -2.34, df = 35.71, p = 0.025) but not at T4 (mean difference, -12.46 mmHg; t = -1.95, df = 31.80, p = 0.061) and T5 (mean difference, -8.08 mmHg; t = -1.70, df = 38.15, p = 0.098). These differences were not statistically significant after Holm adjustment.

For DBP, unadjusted differences were not statistically significant at T0 (mean difference, -4.06 mmHg; t = -0.96, df = 43.53, p = 0.345) and T1 (mean difference, -5.33 mmHg; t = -1.31, df = 45.31, p = 0.196). Lower DBP values in the remimazolam group were observed in the unadjusted analyses at T2 (mean difference, -8.79 mmHg; t = -2.22, df = 39.63, p = 0.032), T3 (mean difference, -10.79 mmHg; t = -2.68, df = 34.03, p = 0.011), T4 (mean difference, -8.35 mmHg; t = -2.58, df = 36.84, p = 0.014), and T5 (mean difference, -6.05 mmHg; t = -2.32, df = 43.04, p = 0.025). These differences were not statistically significant after Holm adjustment.

HR showed no between-group differences from T0 to T5. None of the unadjusted comparisons reached statistical significance (T0: t = 0.82, df = 54.55, p = 0.414; T1: t = -1.03, df = 48.19, p = 0.310; T2: t = -0.67, df = 50.30, p = 0.509; T3: t = -1.08, df = 52.45, p = 0.287; T4: t = -0.63, df = 44.19, p = 0.529; T5: t = -0.27, df = 43.97, p = 0.791).

BIS values were lower in the remimazolam group at several early time points: T0 (mean difference, -7.72; t = -2.16, df = 36.03, p = 0.037), T1 (mean difference, -5.87; t = -2.04, df = 41.45, p = 0.048), T2 (mean difference, -5.18; t = -2.02, df = 47.52, p = 0.049), and T3 (mean difference, -6.29; t = -2.65, df = 56.77, p = 0.011). The differences were not statistically significant at T4 (mean difference, -4.27; t = -1.81, df = 55.16, p = 0.076) and T5 (mean difference, -3.18; t = -1.55, df = 52.83, p = 0.128). These exploratory time-point comparisons were not interpreted as confirmatory after adjustment for multiple comparisons.

**Figure 2:**
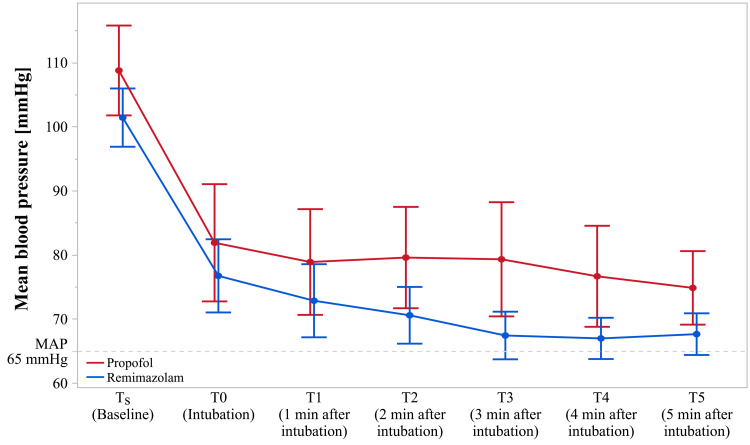
Mean arterial pressure during induction (Ts to T5) Values are presented as means with 95% confidence intervals (CIs) for each time point (Ts = time of hypnotic administration; T0 = tracheal intubation; T1-T5 = one to five minutes after intubation). The dashed horizontal line indicates a mean arterial pressure of 65 mmHg. Two-sided Welch's t tests were performed for mean arterial pressure at each postintubation time point, and the results were adjusted using the Holm method for the six comparisons from T0 to T5.

**Figure 3:**
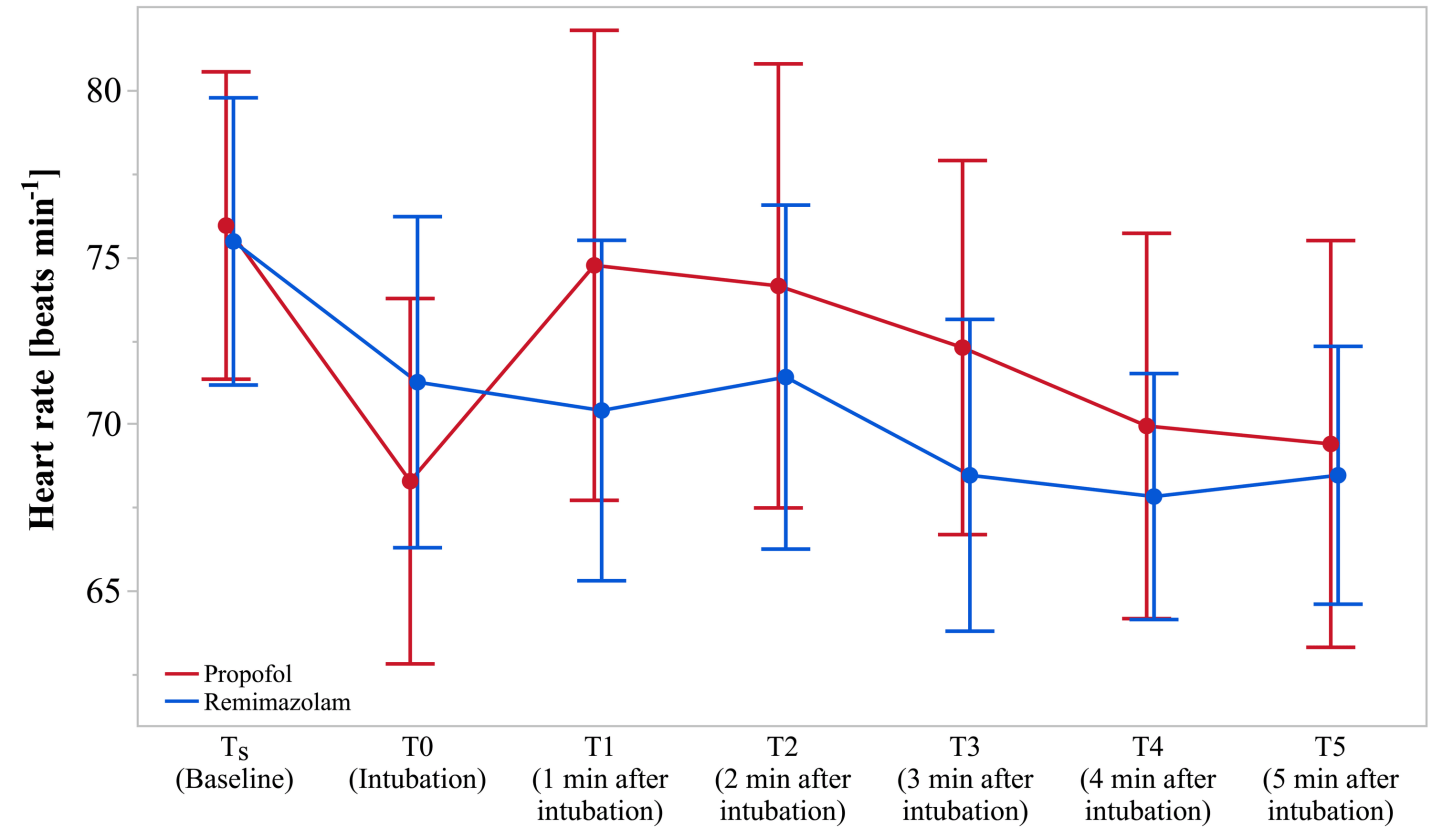
Heart rate during induction (Ts to T5) Values are presented as means with 95% confidence intervals (CIs) at each time (Ts = time of hypnotic administration; T0 = tracheal intubation; T1-T5 = one to five minutes after intubation). Two-sided Welch's t tests were performed for heart rate at each postintubation time point, and Holm adjustment was applied across T0-T5.

The MAP trajectory from Ts to T5 is shown in Figure 2. The HR trajectory over the same period is shown in Figure 3.

## Discussion

In this study, we compared early postintubation hemodynamic changes under a standardized, simultaneous single-bolus protocol using remifentanil with either remimazolam or propofol in patients undergoing THA. The incidence of the primary outcome, Hypo65, did not differ significantly between the remimazolam and propofol groups. Results of sensitivity analyses and comparison of secondary outcomes also showed no consistent significant between-group differences. In the multivariable analysis, older age and lower preinduction MAP were associated with hypotension, whereas hypnotic agent selection was not significantly associated with hypotension.

Remimazolam has been reported to provide more stable hemodynamics than propofol. Phase II/III studies have evaluated standard induction regimens of approximately 0.1-0.2 mg/kg/min (6-12 mg/kg/h), demonstrating noninferiority to propofol [6]. A dose-finding study reported that an infusion rate of 0.10 mg/kg/min achieved loss of consciousness within two minutes in typical adults [15]. In older adults, the reported 90% effective infusion rate is approximately 0.13 mg/kg/min, with limited need for vasopressor or inotropic support during induction in the reported study population [16]. A recent systematic review and meta-analysis also suggested that remimazolam reduces hypotension compared with propofol in older patients undergoing general anesthesia [17]. However, these studies varied in patient populations, dosing strategies, opioid coadministration, and definitions of hypotension.

The rationale of our protocol was to sufficiently suppress sympathetic response to tracheal intubation with remifentanil while using relatively low doses of hypnotic agents, aiming for rapid induction with minimal hemodynamic disturbance. Remimazolam, with its short-acting and highly titratable profile, is expected to cause fewer hemodynamic fluctuations [6]. Therefore, the combination of remifentanil and remimazolam may balance the suppression of intubation response with hemodynamic stability. Although no significant difference was observed between the remimazolam and propofol groups in the present study, no refractory or life-threatening hypotension occurred in either group, and the observed BP reductions were clinically manageable.

In several unadjusted time-point comparisons, MAP, SBP, and DBP tended to be lower in the remimazolam group, although these differences were not statistically significant after Holm adjustment. However, because these trajectory analyses were exploratory and descriptive and did not account for within-patient repeated measurements or vasopressor administration during the T0-T5 period, they should not be interpreted as definitive evidence of comparable hemodynamic profiles. Rather, the present findings suggest that, under this simultaneous-bolus induction protocol, patient-related factors such as age and preinduction BP may be important determinants of early postintubation hypotension, whereas clinically relevant differences between hypnotic agents cannot be excluded. This finding is consistent with previous reports of no clear difference in postinduction BP decline between remimazolam and propofol [7-9].

In the present protocol, a relatively large remifentanil bolus dose (3 μg/kg) was used to sufficiently attenuate the sympathetic response to intubation. Remifentanil bolus doses of approximately 2 μg/kg have been reported to suppress pressor and tachycardic responses to laryngoscopy and tracheal intubation, whereas doses exceeding 1 μg/kg may increase the risk of hypotension [12,13]. Thus, the dose of 3 μg/kg was selected with an emphasis on suppressing the intubation response, but careful consideration of the hypotension risk is required. Importantly, in our protocol, remifentanil was administered as a single bolus rather than as a bolus superimposed on continuous infusion. Because remifentanil is short-acting and highly titratable, circulatory depression after a single bolus may be relatively brief. The absence of refractory or life-threatening hypotension in this cohort suggests that severe hemodynamic instability was not observed under this protocol; however, the small sample size limits conclusions regarding safety.

The present findings suggest that the balance among remifentanil dose, hypnotic dose, injection speed, and patient background is important in simultaneous-bolus induction. In particular, in older patients and those with lower preinduction BP, individualized dose adjustment according to age and baseline hemodynamic status may help reduce hypotension while maintaining rapid induction. Future prospective studies are needed to evaluate the optimal doses and administration methods of remifentanil and remimazolam, as well as individualized induction strategies based on patient characteristics.

Limitations

This study has several important limitations. First, owing to the single-center retrospective design and nonrandom selection of hypnotic agent, residual confounding and allocation bias could not be excluded. The choice of remimazolam or propofol was made by the attending anesthesiologist according to routine clinical practice, and no formal institutional criteria or predefined algorithm mandated the use of either agent. Therefore, clinician preference, perceived patient baseline risk, including age or expected hemodynamic vulnerability, and time-period effects may have influenced group allocation and the observed results. Propensity score matching was not performed because of the limited sample size and the risk of further reducing the analyzable cohort; therefore, residual confounding might have remained.

Second, the small sample size and absence of an a priori sample size calculation contributed to imprecision. The multivariable logistic regression analysis should be interpreted cautiously because the number of outcome events was limited relative to the number of covariates. With 33 outcome events and four covariates in the primary model, the events-per-variable ratio was low, and the model may have been unstable or overfitted. Therefore, the adjusted odds ratios, particularly for hypnotic agent selection, should not be overinterpreted. The CIs for several outcomes were wide, particularly for hypotension-related outcomes and vasopressor use, and remain compatible with clinically relevant between-group differences. Accordingly, the absence of statistically significant differences should not be interpreted as evidence of equivalence or absence of clinically relevant differences between remimazolam- and propofol-based induction.

Third, only 59 of the 190 screened cases were included in the final analysis. Many excluded cases lacked analyzable induction time-series data in the source records, and the predefined T0-T5 hemodynamic and BIS variables could not be reconstructed. Because the required time-point and outcome data were unavailable for most excluded cases, formal comparison between included and excluded patients was not feasible. This high exclusion rate may have introduced selection bias and reduced the generalizability of the findings.

Fourth, the primary outcome was defined as any one-minute epoch with MAP <65 mmHg during T0-T5. Although this binary definition is clinically simple and consistent with commonly used hypotension thresholds, it does not distinguish a brief marginal decrease, such as a MAP of 64 mmHg, from more severe or sustained hypotension. Therefore, the primary analysis may not fully capture the depth, duration, or cumulative burden of hypotension. To partly address this limitation, TWA MAP below 65 mmHg and minimum MAP during induction were evaluated as secondary outcomes. Nevertheless, the primary endpoint should be interpreted as reflecting the occurrence, rather than the severity or cumulative burden, of early postintubation hypotension.

Fifth, this study focused on early postintubation hemodynamics from tracheal intubation to five minutes thereafter. Hypotension occurring before intubation, after T5, or during the subsequent intraoperative period was not included in the primary endpoint. Although preinduction MAP at Ts was recorded as a baseline covariate, hypotension during the interval between hypnotic administration and tracheal intubation was not formally evaluated as a separate outcome. Therefore, the findings should be interpreted as applying specifically to the immediate postintubation period and not to the entire induction or intraoperative course.

Sixth, the hemodynamic trajectory analyses were exploratory and descriptive. MAP, SBP, DBP, HR, and BIS were repeatedly measured within the same patients, but no longitudinal or mixed-effects model was fitted. In addition, vasopressor administration during T0-T5 may have influenced subsequent BP measurements. Although time-point comparisons with Holm adjustment were reported, these analyses were not designed to support definitive inferences regarding detailed hemodynamic trajectories or the comparability of hemodynamic profiles between groups.

Seventh, although the induction sequence, fluid administration, and vasopressor treatment threshold were clarified, exact injection speeds and time intervals between hypnotic administration, rocuronium administration, remifentanil administration, and tracheal intubation were not systematically recorded in the source records. These unmeasured procedural variables may have influenced early postintubation BP values.

Finally, this study included only patients who underwent THA under general anesthesia according to our institutional practice. Therefore, the findings should not be generalized to patients undergoing THA under neuraxial or regional anesthesia, including spinal, epidural, or combined spinal-epidural anesthesia. In addition, because the protocol was applied within a specific institutional practice using a simultaneous-bolus approach, the findings may not be directly generalizable to other induction strategies, including slower remimazolam infusion, different remifentanil doses, or target-controlled propofol administration.

Despite these limitations, the consistency between the primary and sensitivity analyses, together with the evaluation of multiple hemodynamic endpoints, provides internal consistency for the exploratory findings. Prospective studies with standardized data capture are warranted to further evaluate optimal remifentanil dosing and remimazolam administration strategies in patients undergoing THA.

## Conclusions

In this single-center retrospective observational study of patients undergoing THA, no statistically significant difference in early postintubation hypotension within five minutes after tracheal intubation was detected between remimazolam- and propofol-based induction under a standardized simultaneous-bolus protocol with remifentanil. In the exploratory adjusted analysis, older age and lower preinduction MAP were associated with a higher risk of hypotension, whereas hypnotic agent selection was not significantly associated with early postintubation hypotension. However, because of the small sample size, wide CIs, limited events-per-variable ratio, high exclusion rate, nonrandom hypnotic selection, and retrospective design, these findings should be interpreted as exploratory and should not be considered evidence of equivalence or absence of clinically relevant differences between the two induction strategies. Future prospective studies with standardized data capture are warranted to further evaluate individualized induction strategies based on age and baseline hemodynamic status.
